# Comprehensive evaluation of pure and hybrid collaborative filtering in drug repurposing

**DOI:** 10.1038/s41598-025-85927-x

**Published:** 2025-01-21

**Authors:** Clémence Réda, Jill-Jênn Vie, Olaf Wolkenhauer

**Affiliations:** 1 Department of Systems Biology and Bioinformatics, University of Rostock, Rostock, 18051 Germany; 2 Soda Team, Inria Saclay, Palaiseau, 91120 France; 3 Leibniz-Institute for Food Systems Biology, Freising, 85354 Germany; 4 Stellenbosch Institute of Advanced Study, Wallenberg Research Centre, Stellenbosch, 7602 South Africa

**Keywords:** Drug repositioning, Drug repurposing, Collaborative filtering, Benchmark, Matrix factorization, Virtual screening, Computer science, Statistics

## Abstract

Drug development is known to be a costly and time-consuming process, which is prone to high failure rates. Drug repurposing allows drug discovery by reusing already approved compounds. The outcomes of past clinical trials can be used to predict novel drug-disease associations by leveraging drug- and disease-related similarities. To tackle this classification problem, collaborative filtering with implicit feedback (and potentially additional data on drugs and diseases) has become popular. It can handle large imbalances between negative and positive known associations and known and unknown associations. However, properly evaluating the improvement over the state of the art is challenging, as there is no consensus approach to compare models. We propose a reproducible methodology for comparing collaborative filtering-based drug repurposing. We illustrate this method by comparing 11 models from the literature on eight diverse drug repurposing datasets. Based on this benchmark, we derive guidelines to ensure a fair and comprehensive evaluation of the performance of those models. In particular, an uncontrolled bias on unknown associations might lead to severe data leakage and a misestimation of the model’s true performance. Moreover, in drug repurposing, the ability of a model to extrapolate beyond its training distribution is crucial and should also be assessed. Finally, we identified a subcategory of collaborative filtering that seems efficient and robust to distribution shifts. Benchmarks constitute an essential step towards increased reproducibility and more accessible development of competitive drug repurposing methods.

## Introduction

Developing novel drugs has turned out to be a long, strict and therefore costly process. The time window between identifying a drug candidate and its marketing is around 5 years, but it can take 10 years and cost an average of $2.3 billon^1^. Still, the failure rate in commercializing a candidate drug is up to $$90\%$$^2^. This has led researchers to consider already well-understood drugs instead of de novo drug designs.

Drug repurposing aims to screen large libraries of well-documented chemical compounds in an automated fashion to uncover new drug-disease associations. This is supported by the availability of clinical (trial) data^3^, omics data from drug perturbations^4^, drug sensitivities^5^, as well as databases providing details of molecular structures and chemical properties. The rise of machine learning approaches and increasing computational power have raised the interest in drug repurposing.

The underlying hypothesis behind drug repurposing is that drug molecules can target multiple biological processes in which dysregulations are causal factors accounting for a given pathology. Diseases might share those dysregulations^6^. Moreover, since drug discovery is restricted to approved molecules, drug repurposing speeds up the early preclinical phases and toxicity analyses in the pipeline. Focusing on well-known molecules, in turn, could reduce the risk of unexpected adverse side effects at late development stages, which still constitute one of the main reasons for marketing failure in late clinical phases^7^.

Several approaches to drug repurposing have been developed in the literature. We refer to^8,9^ for a comprehensive overview of those methods. In drug repurposing, a classifier can be trained to match and predict outcomes from past clinical trials, as made available by ClinicalTrials.gov^3^, or the RepoDB database^10^ for instance. Such a classifier might be based on relevant biological features of drugs and diseases, or rely solely on the reported clinical trial outcomes. Those outcomes are known to be highly imbalanced between positive and negative outcomes because negative results are rarely reported^11,12^. Those adverse outcomes might result from late discovery toxicity effects or low accrual. Moreover, the number of untested drug-disease associations dramatically outnumbers the number of past clinical trials. For example, in the TRANSCRIPT^13^ and PREDICT^14^ datasets which were published last year, the ratio between negative and positive drug-disease matches is around $3\%$. In contrast, the sparsity number—the percentage of unknown matches over the total number of possible matches—is larger than $98.5\%$. Attempting to overcome this lack of data by considering all unknown outcomes as negative, as tempting as it may be, might induce considerable bias in the underlying model. Indeed, a drug-disease association might not have been tested for various reasons, including the incompleteness of knowledge on biological events. This might explain that binary classifiers fail on not fully annotated datasets^15^. Moreover, another reason untested drug-disease pairs cannot be considered fully-fledged negative results is that one is looking for novel drug indications among these pairs. Nonetheless, the fact that a drug-disease association has not been tested is already informative. This type of implicit information (often named *implicit feedback*) arises in many other non-medical topics of recommendation, for instance advertising^16^.

**Fig. 1:**
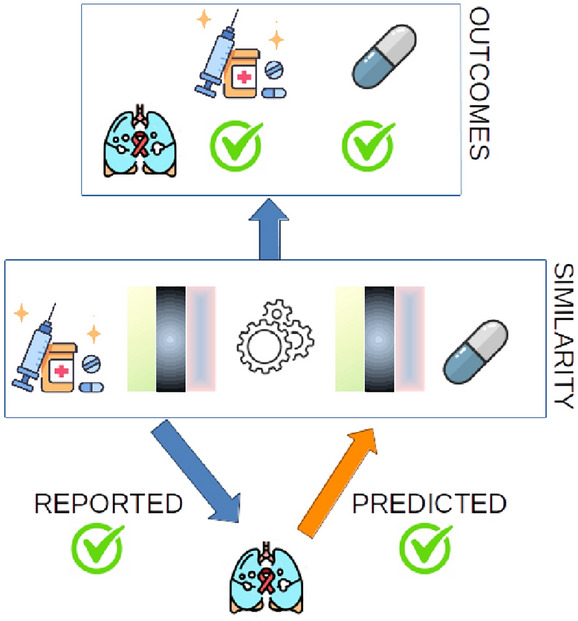
Principle of collaborative filtering. If two drugs A and B are similar, and if there is a known association between a disease and drug A, then the same association is predicted between this disease and drug B.

Collaborative filtering is a flexible semi-supervised approach that has raised a lot of interest in the domain of recommendation systems. This framework has also become popular in drug repurposing, considering drugs as items and diseases as users^17,18^, notably thanks to the Netflix Prize problem^19^, which aimed to connect movies and viewers. Predicted drug-disease associations stem from a function whose parameters are learned on a whole matrix of drug-disease matches instead of focusing on a single disease at a time. Then, such methods rely on filtering patterns learned across diseases and drugs, implementing some collaboration (see Fig. 1 for an illustration of this principle). A few examples of simple collaborative filtering methods are nearest neighbor approaches, where an outcome is assigned to a pair based on a consensus on similar datapoints^20^, and matrix factorization, in which literature often relies on tensor decomposition, *i*.*e*., any drug-disease matching in the matrix is the output of a classifier in which only lower-rank tensors intervene. This principle is present, for instance, in factorization machines^21^. For those algorithms, the classifier may only take as input the matrix of drug-disease associations (*pure* collaborative filtering). *Hybrid* collaborative filtering relies on supplementary features for drugs and diseases in addition to the association matrix. Those features might be similarity scores across diseases and drugs or experimental measurements.

Although the application of collaborative filtering to drug repurposing has become increasingly popular in the last 10 years^16–18,22–26^, the field lacks a standard benchmark approach to evaluate the performance of new algorithms. Across papers, several different metrics, datasets, and baseline algorithms have been selected, undermining the comparability and application of the proposed methods. Due to the hurdles in running the methods and accessing drug repurposing datasets, numerical results from baseline algorithms are sometimes copied directly from the original paper. Moreover, reproducibility issues specific to the implementation of the experiments further undermine the experimental results: for instance, not setting a fixed random seed, varying number of iterations, lack of package versioning, and differences in hyperparameter tuning. As a general rule, such a reproducibility issue is still pervasive in machine learning, as raised by several papers^27–29^. Conversely, the tremendous progress in computer vision and large language models (LLMs), for instance, has been credited to constructing standard datasets and benchmarks in those fields^30,31^.

**Contributions** **1.** To bridge that gap in the literature, we performed a benchmark across 11 published and open-access drug repurposing approaches based on collaborative filtering (see Table 2) on eight different drug repurposing datasets and a synthetic one (see Table 1). The algorithms and the datasets are available *via* two recently published open-source Python packages^32^. **2.** This large-scale benchmark allowed us to suggest guidelines for performing a fair and comprehensive assessment of those methods applied to drug repurposing. In particular, the dataset selection, the validation metric, and the split into training and testing sets are crucial to a benchmark. **3.** We show that methods relying on constructing a heterogeneous graph connecting drugs and diseases usually perform best in this benchmark. This result will hopefully support the faster development of novel approaches to drug repurposing, especially regarding interpretability.

In the following sections, we formally define the drug repurposing problem in a collaborative filtering framework and suggest a classification of state-of-the-art algorithms that tackle this problem. In the problem statement, we describe the methodology behind our benchmark, along with the selected algorithms and datasets. The experimental study displays our benchmark results, that is, the ranking of the considered state-of-the-art algorithms and the experiments specific to the choice of a dataset and a validation metric.

## Results

### Problem statement

Part of our contribution to this work is an overview of state-of-the-art approaches to collaborative filtering, especially in drug repurposing. We also provide insights into applying these algorithms for medical and biological research.

**Table 1:** Datasets in the benchmark. They correspond to the number of drugs and diseases involved in at least one nonzero drug-disease association. The sparsity *s* is the percentage of unknown (neither positive nor negative) matches times 100 over the total number of possible drug-disease matches (rounded up to the first decimal place). The imbalance ratio **IR** is the ratio between negative and positive outcomes in the dataset (rounded up to the second decimal place). The private version of PREDICT is the one generated from notebooks in the original GitHub repository, whereas the public one is the one deposited on Zenodo^14^. The association matrix in the Fdataset comes from^34^. Still, the drug and disease features are from^33^.

Type	Dataset	Paper	$N_S$	$F_S$	$N_P$	$F_P$	#Positive	#Negative	*s* (%)	IR ($\%$)
Text-mining	Cdataset	^33^	663	663	409	409	2,532	0	99.1	0
	Fdataset	^33,34^	593	593	313	313	1933	0	99.0	0
	DNdataset	^35^	550	1490	360	4516	1008	0	99.5	0
Biological	Gottlieb	^34,36^	593	1779	313	313	1933	0	99.0	0
	LRSSL	^37^	763	2049	681	681	3051	0	99.4	0
	PREDICT	^14^	1351	6265	1066	2914	5624	152	99.6	2.70
	PREDICT	^14^	1014	1642	941	1490	4627	132	99.5	2.85
	TRANSCRIPT	^13^	204	12,096	116	12,096	401	11	98.3	2.74
Artificial	Synthetic	^32^	300	25	300	25	200	100	99.7	50

#### The drug repurposing problem  

A drug repurposing dataset comprises a drug-disease association matrix denoted $A \in \{-1,0,+1\}^{N_S \times N_P}$, which summarizes all known matches between chemical compounds and pathologies. $N_S$ is the number of drugs, and $N_P$ is the number of diseases for which at least one matching with a disease/drug is known. That is, every row and every column in matrix *A* has at least one non-zero coefficient. 0 means that the drug-disease association is deemed unknown (for instance, no Phase III clinical trial testing of this association has been reported). $+1$ means that the drug is efficient in treating the disease, for instance, through a successful clinical trial. $-1$ means that matching the drug and the disease is not recommended. Notably, until recently, no drug repurposing datasets featured negative associations (see Table 1) due to the difficulty in defining a negative association, and only comprise positive or unknown associations. In the remainder of this paper, similarly to a prior work^32^, we define a negative drug-disease association as a drug-disease pair where either the drug is too toxic or too inefficient (*e*.*g*., linked to reported low accrual in clinical trials). We expect those explicit negative annotations to improve the performance of a drug repurposing classifier outputting labels in $\{-1,+1\}$. How to take into account negative examples is still the subject of recent theoretical works on collaborative filtering^38^, but it has not been tackled in the applications to drug repurposing. Ultimately, collaborative filtering aims to replace zeroes in matrix *A* by values in $\{-1,+1\}$. In the remainder of the paper, we denote $\hat{R} \in \mathbb {R}^{N_S \times N_P}$ the predicted association score matrix.

In addition to the association matrix *A*, some information about the drugs and diseases is also available to define drug and disease similarities. That information might be used by hybrid collaborative filtering algorithms. Different data types are featured in currently available drug repurposing datasets, as shown in Table 1. Drug and disease feature information is very heterogeneous: for instance, the Cdataset, the Fdataset^33^, and the DNdataset^35^ rely on text-mining approaches. More specifically, the drug-disease associations are first mined from the DrugBank^39^ database. Then, for the Cdataset and Fdataset, the drug information *S* corresponds to Tanimoto drug similarity scores computed on 2D fingerprints of chemical structures. In contrast, disease features in *P* are a disease similarity matrix computed on their respective medical descriptions in OMIM^40^. In DNdataset, the drug similarity matrix *S* is computed using Lin’s node-based similarity function^41^ on the anatomical therapeutic chemical (ATC) codes for drugs. Lin’s node-based similarity is also applied to disease ontologies^42^ for the disease similarity matrix *P*. Note that those similarities are computed on a set of drugs and diseases larger than the number of entities involved in at least one non-zero association.

Recently, some works proposed biological data-based datasets for collaborative filtering-based drug repurposing. In the LRSSL dataset^37^, drug features include the binary fingerprints of chemical structures and target protein domains and disease features are disease semantic similarities based on the intersection between disease-specific directed acyclic graphs of descriptors^43^. Similarly, the Gottlieb dataset^36^ comprise drug-pairwise chemical, domain, functional (as Jaccard scores computed on Gene Ontology^44^) and disease semantic similarity matrices on drugs and diseases present in the associations in Fdataset. Those similarity matrices are concatenated in Table 1. The PREDICT^14^ dataset incorporates several types of drug and disease similarity measures based on disease phenotypes, drug chemical structures, target gene proximity in a protein-protein interaction network, etc., similar to what was described in the seminal paper of the PREDICT method^34^. Finally, the TRANSCRIPT^13^ dataset only includes transcriptomic-related data, as the drug and disease features are variations of gene-wise transcriptomic levels induced by the corresponding treatment/pathology, computed by performing a differential analysis on relevant samples from the LINCS L1000 database^4^ (for drugs) or retrieved from the CREEDS database^45^ (for diseases and drugs missing from LINCS L1000). Note that the code that generated both datasets is open-source^46^.

All of that drug (resp., disease)-related information is summarized in a drug and a disease feature matrices $S \in \mathbb {R}^{N_S \times F_S}$ and $P \in \mathbb {R}^{N_P \times F_P}$. $F_S$ is the number of drug features (*e*.*g*., genes when considering gene expression data, drugs when *S* is a similarity matrix), and analogously, $F_P$ is the number of disease features. When not considering features, collaborative filtering relies on drug-drug and disease-disease similarities by comparing rows and columns of matrix *A*. For instance, if drug *d* is associated with row $r_d = [+1,0,+1,-1]$ in matrix *A*, and drug $d'$ with row $r_{d'} = [+1,+1,+1,-1]$, then we can possibly set the second coefficient of $r_d$ to $+1$. Note that we ignore in this work the impact of missing and non-finite values on classification, *e*.*g*., $S \in (\mathbb {R}\cup \{\pm \inf , \texttt {N/A}\})^{N_S \times F_S}$, which is in practice extremely relevant when dealing with real-life data. See the methods for the processing of non-finite data. Information about the overlaps between the drug repurposing datasets is available in the methods.

**Table 2:** Overview of algorithms present in the benchmark and the classification (columns “Class” and “I/O type”) defined in the problem statement section.

Class of algorithms	Name	Paper	I/O type	Hybrid	Implementation
Matrix factorization	ALS-WR	^47^	Matrix	$\times$	Python
	LibMF	^48^	Matrix	$\times$	Python
	LogisticMF	^49^	Matrix	$\times$	Python
	PMF	^50^	Matrix	$\times$	Python
	SCPMF	^51^	Matrix	$\times$	MATLAB / Octave
Neural Network	Fast.ai collab_learner	^52^	Pair	$\times$	Python
	NIMCGCN	^53^	Pair	$\checkmark$	Python
Graph-Based	BNNR	^18^	Matrix	$\checkmark$	MATLAB / Octave
	DRRS	^54^	Matrix	$\checkmark$	MATLAB Compiler
	HAN	^55^	Pair	$\checkmark$	Python
	LRSSL	^37^	Pair	$\checkmark$	R

#### Classification of collaborative filtering algorithms  

Based on our review of the literature in the domain in Table 2, we define three large classes of algorithms that depend on the underlying mechanism of repurposing.

Matrix factorization algorithms typically ignore side information from matrices *S* and *P* and aim to infer low-rank tensors such that a function of their product is as close as possible to matrix *A*. As such, these algorithms take the incomplete association matrix *A* as primary input and output the “completed” matrix $\hat{R} \in \mathbb {R}^{N_S \times N_P}$ which should match *A* on its known coefficients. High scores in $\hat{R}$ should match positive coefficients in *A*, and conversely, low scores should correspond to negative or null values in *A*. Predictions on unknown drug-disease matches are made by setting a threshold *t* on the scores, such that drug-disease pair (*i*, *j*) is a positive association if and only if $\hat{R}_{i,j}>t$.

Neural networks are versatile algorithms that can be applied to classification. Given a set of weights $\theta$, a neural network *f* defines the outcome associated with a feature vector *x* of a drug-disease pair by $f_\theta (x) \in \mathbb {R}$. Again, such outcomes should match the values in *A*. One might obtain true labels either by a thresholding approach or by adding a last softmax layer to the network and outputting the class associated with the highest score. However, contrary to most matrix factorization approaches, neural networks are a flexible way to integrate supplementary information about drugs and diseases in matrices *S* and *P* or to learn embeddings of drugs and diseases based on shared matches.

Finally, we define a third, less obvious class of algorithms called “graph-based”. Albeit they might rely to some extent on neural networks and tensor factorization, they are characterized by their building of a heterogenous (not necessarily bipartite) graph connecting drugs and diseases. Often, the edges of this graph can be split into three main groups: edges connecting a pair of drugs, a pair of diseases, or a drug and a disease. Drug repurposing aims to reconstruct edges from the last set, but a critical side advantage of those algorithms is to retrieve similarities between drugs and diseases. In particular, such edges might be helpful to justify predicted drug-disease associations and contribute to the interpretability of classifiers. This algorithm can either output pair-related scores or a full association matrix (see Table 2).

#### Pairs or matrices?  

In addition to the three classes of algorithms defined in the last paragraph, state-of-the-art algorithms can also be discriminated by the type of their input/output (column “I/O type” in Table 2). In particular, those algorithms receive and output either a drug-disease association matrix or a drug-disease pair. We emphasize that choosing one type of algorithm or the other considerably impacts the resulting repurposing, both at training and prediction times. We would not recommend using matrix-oriented methods in drug repurposing.

Indeed, at training/testing time, when run on a subset of a drug repurposing dataset, algorithms that take as input an entire matrix cannot distinguish between “accessible” zeroes of the association matrix (*i*.*e*., zeroes in the whole, initial, drug repurposing dataset) and “inaccessible” zeroes (that is, drug-disease matches which are masked in the subset but are non-zero coefficients in the full dataset). This simultaneously leads to data leakage and corrupted validation.

The data leakage stems from the fact that, in that case, an unknown drug-disease matching can never be hidden in the training set, as there is no mechanism to encode “inaccessible” *true* zeroes in the association matrix. As such, the algorithm is trained on information that is supposed to be accessible only at testing time. An approach to avoid this would be to ensure all zeroes in the initial association matrix *A* belong to the training set and none belong to the validation subset. Then, the chosen accuracy metric would be computed only on non-zero elements of the validation subset. Since most drug repurposing datasets only feature 0–1 values (and none of the true negatives denoted by $-1$’s), most standard metrics cannot be computed, as they require at least two types of labels. That metric type notably includes the popular Area Under the Curve (AUC). Note that, given the (very) low number of negative drug-disease associations in Table 1, restricting the training to datasets involving at least one negative example would inevitably lead to overfitting, which is, of course, undesirable. This problem of data leakage cannot then be fixed and might, unfortunately, account for the apparent good results of matrix-oriented approaches in our benchmark (see the experimental study).

The corrupted validation comes from an incorrect implementation of the validation procedure, which is present in papers mentioning matrix-oriented approaches for drug repurposing and publishing code for their experiments. Indeed, if the selected accuracy/validation metric is computed across all coefficients/labels of matrix $\hat{R}$, regardless of the accessibility of the coefficients at training time, this metric might be inflated by the values obtained on unknown drug-disease pairs. This issue was solved during the implementation of our benchmark. Indeed, regardless of the input type of the benchmarked algorithm, the validation metrics are computed on a *fold* and never directly on the predicted and ground truth association matrices $(\hat{R}_{i,j}, A_{i,j})_{i \le N_S, j \le N_P}$. A fold is defined as a set of values referring to drug-disease pairs: *i*.*e*., a set of indices $\mathcal {I} \subseteq \{1,2,\dots , N_S\} \times \{1,2,\dots , N_P\}$ such that the validation metric is computed on vectors $(\hat{R}_{i,j}, A_{i,j})_ {(i,j) \in \mathcal {I}}$.

Moreover, at prediction time, matrix-oriented approaches can only provide predictions for drugs and diseases present in the matrix on which they have been trained. Suppose one needs to predict the outcome of a new drug-disease pair. In that case, one needs to concatenate information about this new drug or disease to the initial association matrix, run a training routine on this matrix again, and then make predictions. The same goes for supplementary information about drug-disease matches accrued after the initial training of the model. Consequently, this is potentially time-consuming and hinders drug repurposing of novel compounds.

Note that there are already a vast literature on biases in collaborative filtering, which are related to unknown associations: for instance, the exposure bias^56^ (users are exposed to few items, so unknown does not necessarily mean negative), the popularity bias (items most frequently interacted with in the training set are more frequently recommended), the not missing at random bias^57^ (an association label might be missing due to an unobserved latent factor), and many others^58^.

However, we are the first to alert on the issue arising from not distinguishing between zeroes in the training set (unknown associations in the dataset) and the mask that zeroes out any value in the testing set (needed in what we call “matrix-oriented” algorithms) in drug repurposing. Contrary to all other previously mentioned biases, this bias is not linked to implicit feedback in the data, but to the structure of the recommender system (matrix or pair-oriented). As such, debiasing techniques present in the literature (*e*.*g*., using propensity scores^59^, sampling or causal learning approaches^58^) are not appropriate to deal with this specific evaluation bias.

**Table 3:** Description of the considered validation metrics present in the benchmark. $\Omega ^\pm \triangleq \{(i,j), A_{i,j} = \pm 1 \mid i \le N_S, j \le N_P\}$ is the set of all positive ($\Omega ^+$) or negative ($\Omega ^-$) drug-disease associations, whereas $\Omega ^+_j \triangleq \{i \mid A_{i,j} = +1\}$ is the set of drugs involved in positive associations with disease *j* and $\widetilde{\Omega }_j \triangleq \{(i,i') \mid A_{i,j} > A_{i',j}\}$ for any $j \le N_P$ is the set of correctly ordered pairs of drugs for the score ranking in disease *j*. In the benchmark, $t=0$ and $\mathbbm {1}(C)$ is equal to 1 if *C* is satisfied, 0 otherwise. $\sigma _{V}$ is the permutation that sorts all coefficients of any vector *V* of length *n* in decreasing order, that is, $V_{\sigma _V(1)} \ge V_{\sigma _V(2)} \ge \dots \ge V_{\sigma _V(n)}$. The true positive rate is formally defined as $\texttt {TPR}(t; \hat{R}, A) = \sum _{(i,j),A_{i,j}=+1} \mathbbm {1}(\hat{R}_{i,j}>t)/\sum _{(i,j)} \mathbbm {1}(\hat{R}_{i,j}>t)$ and $\texttt {FPR}(t; \hat{R}, A) = \sum _{(i,j),A_{i,j}=-1} \mathbbm {1}(\hat{R}_{i,j}>t)/\sum _{(i,j)} \mathbbm {1}(\hat{R}_{i,j}\le t)$ is the false positive rate. Finally, $N^{+,j}_S$ is defined as $\min (N_S,|\Omega ^+_j|)$.

Type	Metric	Notation	Formula
Global	Accuracy	$\texttt {Acc}(\hat{R},A;t)$	$(|\Omega ^-|+|\Omega ^+|)^{-1} \sum _{(i,j) \in \Omega ^- \cup \Omega ^+} \mathbbm {1}((\hat{R}_{i,j}-t)A_{i,j} > 0)$
	Area Under the Curve	$\texttt {AUC}(\hat{R},A)$	$\int _0^1 \text {TPR}(\text {FPR}^{-1}(x; \hat{R}, A); \hat{R}, A)dx$
Local	Average AUC	$\texttt {AUC}_d(\hat{R},A)$	$N_P^{-1} \sum _{j \le N_P} \texttt {AUC}(\hat{R}[\cdot ,j], A[\cdot ,j])$
	Average NS-AUC^60^	$\texttt {NS-AUC}(\hat{R},A)$	$|N_P|^{-1} \sum _{j \le N_P} |\widetilde{\Omega }_j|^{-1} \sum _{(i,i') \in \widetilde{\Omega }_j} \mathbbm {1}(\hat{R}_{i,j}>\hat{R}_{i',j})$
	Average NDCG@$N_S$	$\texttt {NDCG}(\hat{R},A)$	$N_P^{-1} \sum _{j \le N_P} \left( \sum _{i=1}^{N^{+,j}_S} \frac{A_{\sigma _{\hat{R}_{\cdot ,j}}(i), j}}{\log _2(i+1)} \right) /\left( \sum _{i=1}^{N^{+,j}_S} \frac{1}{\log _2(i+1)} \right)$

#### Validation metrics for drug repurposing

As illustrated by Table 1, drug repurposing datasets are highly imbalanced and information-scarce, both between the known ($-1/+1$) and unknown (0) labels (column “sparsity”), and between the positive ($+1$) and negative ($-1$) associations (column “IR”). As such, a standard accuracy metric that only accounts for correct label predictions on known drug-disease associations is bound to be biased^61^. Moreover, only focusing on binary labels removes essential information about the ability of the model to rank drug-disease associations. We suggest several conditions to get the best interpretation out of a validation metric (in particular, for real-life applications). The metric should be bounded, ideally in the range [0, 1], where 1 applies to a perfect drug repurposing model, 0 to a model which perfectly ranks *negative associations first*, and finally 0.5 for a ranking at random. See Table 3 for a few examples of standard metrics that satisfy these constraints.

The global accuracy (ACC) is the number of correctly predicted associations over total known (positive and negative) associations. The global Area Under the Curve (AUC) is the area under the curve when plotting the true positive rate against the false positive rate for different thresholds for labeling classes from all scores. The local AUC is the area under the curve at a fixed disease. The local metric Negative Sampling-AUC (NS-AUC)^60^ is the frequency of correctly ranked drug pairs at a fixed disease. For instance, drug $d_1$ is positively associated with disease D, and the indication of drug $d_2$ for disease D is negative or unknown. Then a good classifier should rank the association ($d_1$, D) before ($d_2$, D). An illustration of the NS-AUC is provided in the methods. Finally, the Non-Discounted Cumulative Gain (NDCG) for a fixed disease at rank $N_S$ is the similarity of the ranking given by the classifier up to rank $N_S$ to a perfect ranking of all drugs (putting all positive drug-disease pairs first). We obtain the average value of local metrics by averaging across diseases.

Moreover, in the application of drug repurposing, given that some diseases are investigated more than others, there is a discrepancy in the amount of information available on diseases. This is why we distinguish in Table 3 between “global” metrics, computed across all associations, and “local” ones, which average the metric obtained on disease-specific associations. As we will show in our benchmark, models aiming at optimizing a global metric will not necessarily maximize a local metric.

As a consequence, we conjecture that a model that achieves a high global validation metric on a training set might provide a degraded prediction for a specific disease. This situation would not be satisfying for drug repurposing.

#### Quantifying robustness  

In addition to the evaluation of the approximation error of a model—that is, how well the model retrieves known drug–disease associations—one is also interested in quantifying the robustness of the model and checking whether the model still performs well on data which is significantly dissimilar from the training data. This problem is pervasive in machine learning, particularly in health-related applications^62^, where differences in technicians and measurement protocols can induce a shift in the distribution of values in the data. In prior works^34^, this robustness was measured by training and testing a model on two datasets such that the Tanimoto score between one drug in the training set and another drug in the testing set is at most equal to 0.8.

In our benchmark, we generalize this procedure to other data types than structural fingerprints by splitting in an automated dataset into *weakly correlated* subsets depending on the drug similarity, as described in the methods. This procedure allows us to have a proxy of the error induced by the distribution shift between the training and testing sets.

### Experimental study

**Fig. 2:**
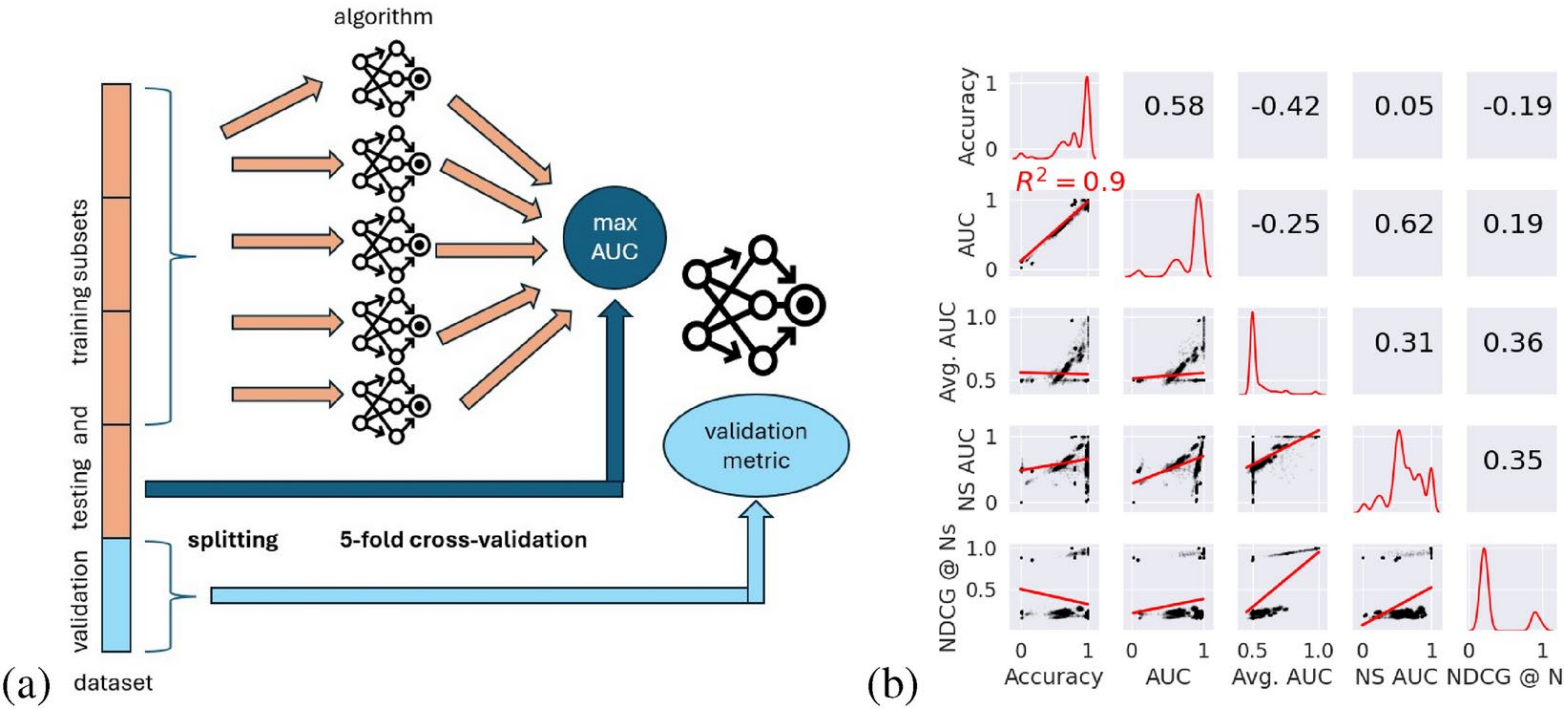
(**a**) Benchmarking training and testing pipeline iterated $N=100$ times for drug repurposing for a specific algorithm, a splitting method for training/testing and validation subsets, and a validation metric. Note that the training/testing subsets are always split at random. (**b**) Correlogram of metrics collected during the benchmark on randomly split training and testing sets, referring to metrics in Table 3. The total number of considered values is then $N=18,700$ (see Table 9 in Appendix). The lower triangle of the plot shows linear regressions between each pair of metrics, with the corresponding $R^2$ when greater than 0.25. The upper triangle displays the Spearman’s $\rho$ correlations between each pair of metrics. The diagonal shows the empirical frequency distribution of values for each metric.

**Fig. 3:**
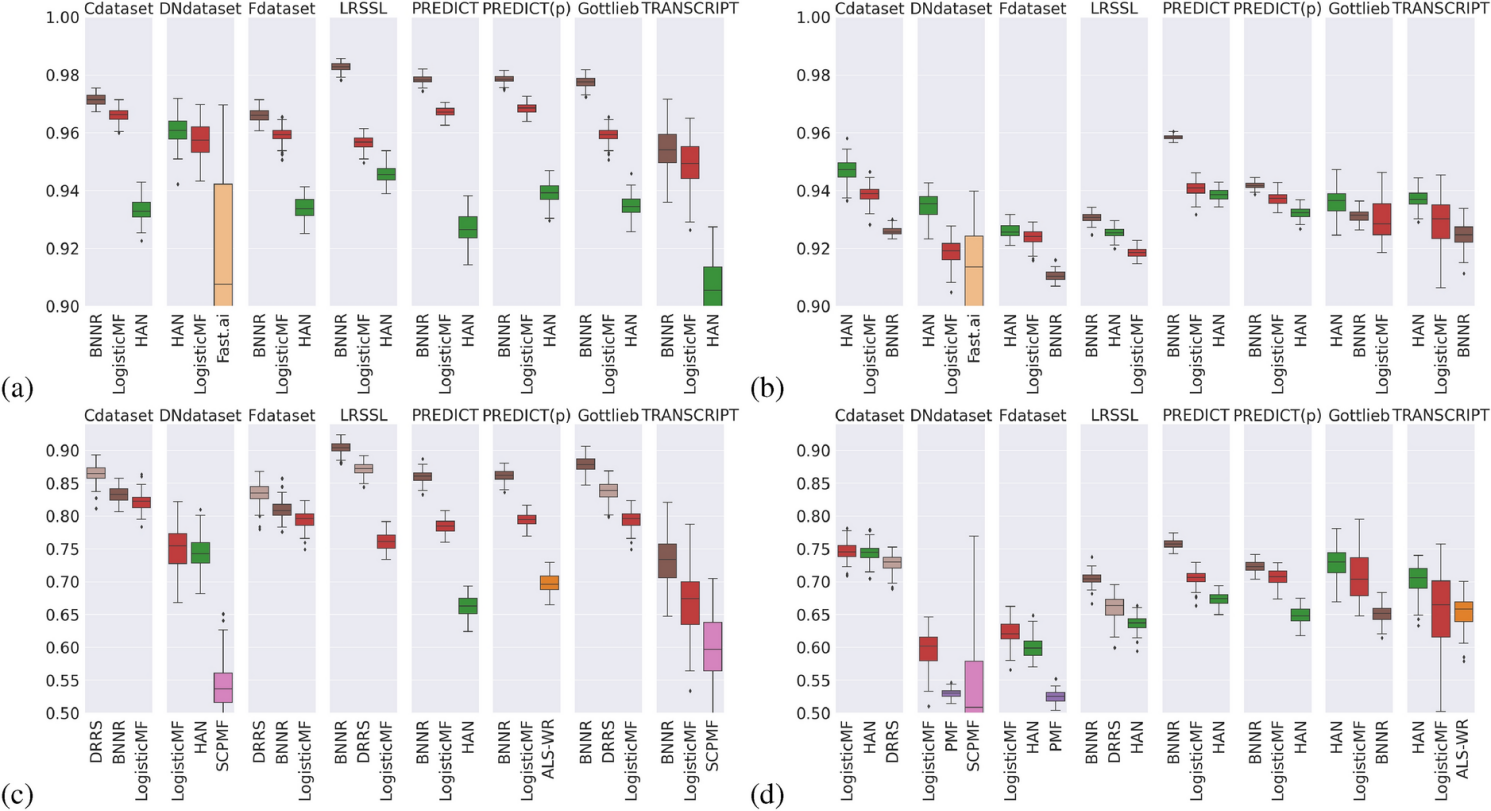
Boxplots of testing metric values for the Top-3 algorithms (in average) across $N=100$ iterations for each dataset in Table 1, for a specific training/testing set splitting method. PREDICT(p) corresponds to the public version of PREDICT, whereas PREDICT refers to the private version of the dataset. (**a**) AUC values for randomly split sets. (**b**) AUC values for weakly correlated sets. (**c**) NS-AUC values for randomly split sets. (**d**) NS-AUC values for weakly correlated sets.

We ran $N=100$ iterations of each algorithm in Table 2 on each dataset in Table 1, and collected all metrics present in Table 3 as computed on the testing subset ($20\%$ of the total dataset) with the best model selected through a 5-fold cross validation on the training subset. The best model is the one that achieves the highest value of AUC across all five folds. Unless otherwise specified, a dataset is randomly split into training and testing sets containing disjoint drug–disease pairs. Figure 2a shows a summary of the benchmarking pipeline. We summarize our insights from the benchmark in Table 4, highlighting the main research questions and our suggestions for tackling each of them. Figure 3 is the crucial result of the benchmark and shows the Top-3 contenders (in terms of average testing accuracy metric) for each dataset. We first consider questions regarding the evaluation of the drug repurposing performance.

**Table 4:** Our guidelines for fairer and comprehensive benchmarks of collaborative-filtering-based drug repurposing models. MF: matrix factorization. NN: neural network. GB: graph-based.

Topic	Questions	Our recommendation
Evaluation of models	**RQ1.** Which metric should the model optimize for?	NS-AUC
	**RQ2.** Which dataset should the model be evaluated on?	PREDICT (*private*) or DNdataset
Future models	**RQ3.** Should a method be pair- or matrix-oriented?	Pair-oriented
	**RQ4.** Which type of algorithms (MF, NN, GB) is the most promising?	Graph-based

#### Optimizing for AUC does not guarantee good disease-wise, nor ranking performance  

We chose to perform model selection based on optimizing the (global) AUC, as done in many prior works^33,34^. Figure 2b compares the distribution of the different metrics in Table 3. Unsurprisingly, as the models run on the testing subsets are selected based on their AUC value on the validation subset (part of the training subset), the AUC and accuracy values obtained on the testing subsets are overall relatively high. However, as illustrated by the diagonal plots and correlation values in Fig. 2b, AUC is only weakly positively correlated to local metrics (average AUC, average NS-AUC) and ranking metrics (NDCG$@N_S$). This is also illustrated in Fig. 3 where the Top-3 algorithms in average testing AUC often differ from those computed based on average NS-AUC values (in 12 out of 16 comparisons). In the context of drug repurposing, the typical use case is to consider a disease for which treatments are missing (*e*.*g*., in rare diseases) or no longer as effective (*e*.*g*., in refractory epilepsies) and predict new therapeutic indications for this disease from a drug library. The first answer to RQ1 in Table 4 (“Which metric should the model optimize for?”) would be NS-AUC. On the other hand, users of a drug repurposing method might also be interested in a good ranking performance, as typically, several drug candidates will be outputted and checked in decreasing order of the associated scores. In that case, the answer to RQ1 would be NDCG$@N_S$.

#### Negative-sampling AUC (NS-AUC) is a good measure of the performance of a model  

^60^ introduced what we call the “negative-sampling AUC” metric , which corresponds to the percentage of the natural order of associations (positive associations first, negative ones last, separated by unknown pairs) which is preserved by a classifier. The full expression of this metric is displayed in Table 3. Compared to the ranking measure NDCG$@N_S$, the NS-AUC has the advantage of being more strongly positively correlated with a global performance on known and unknown pairs (accuracy and “global” AUC values), as exemplified by Fig. 2b. Ultimately, the answer we recommend to Question 1 is to optimize for NS-AUC when training a drug repurposing model, as it fits the drug repurposing use case and obtains good performance for other validation metrics. Based on this recommendation, we focus on NS-AUC values to draw our conclusions in the remainder of this paper.

**Table 5:** Median NS-AUC value across Top-3 algorithms (in average) and all $N=100$ iterations for each dataset in Table 1. The values are rounded up to the closest second decimal place.

Synthetic	LRSSL	Gottlieb	Cdataset	Fdataset	PREDICT (*public*)	PREDICT (private)	DNdataset	TRANSCRIPT
1.00	0.87	0.84	0.84	0.81	0.79	0.78	0.73	0.68

#### There is a need for more diverse reference drug repurposing datasets  

The next question in Table 4 is “Which dataset should the model be evaluated on?”. In a benchmark of drug repurposing approaches, a reference dataset should feature data types that can be retrieved from public databases in a real-life application and be challenging enough to discriminate between drug repurposing algorithms. To quantify the difficulty associated with a dataset, we computed the median NS-AUC value across the Top-3 algorithms in average and all $N=100$ iterations for this specific dataset. We focused on the top-3 contenders to determine a proper baseline for the performance expected on this dataset. The datasets are ranked according to these resulting scores in Table 5. As a sanity check, the synthetic dataset that we have built is indeed very easy, as more than 50% of the time, the best algorithms on this dataset achieve perfect predictive power. The most frequent datasets present in the literature (LRSSL^36,37^, Cdataset, Fdataset^54^) also come at the top of this ranking, which seem unsurprising as most of the state-of-the-art algorithms which we have considered in the benchmark were trained (and probably finetuned) on these datasets. Moreover, these datasets are among the less sparse across all drug repurposing datasets according to Table 1. More interestingly, as described in the problem statement, those datasets share the same types of data, namely, drug–disease associations from DrugBank, drug-pairwise chemical structure similarities, and disease-pairwise semantic similarities. This might explain why, even if they haven’t been tested on all of these “silver standard” datasets, state-of-the-art algorithms generally perform well on these. However, the DNdataset featuring drug annotation codes and disease ontologies, along with the newer PREDICT and TRANSCRIPT datasets with supplementary information from transcriptomics and regulatory networks, is a lot more challenging, as evidenced by the apparent drop in the ranking score. Note that even though there seems to be a correlation between low association sparsity and higher recommendation performance, the TRANSCRIPT dataset is the least sparse of all datasets ($s < 99\%$) and yet also the hardest one. Then, we consider that the new challenge in drug repurposing is to beat the state-of-the-art on these three datasets.

#### Biological data-based drug and disease features are predictive of drug–disease associations  

However, perhaps the three datasets DNdataset, PREDICT and TRANSCRIPT have low ranking scores in Table 5 because the corresponding drug and disease features are not predictive of the drug–disease associations, hence inducing into error most of the drug repurposing algorithms. To test this theory on these three datasets, we used a (non-parametric) Kruskal–Wallis H-test to check whether the NS-AUC median value obtained with feature-agnostic algorithms was significantly different (and greater) than the NS-AUC median value obtained with algorithms that take into account drug and disease features. At significance level $\alpha =1\%$ and adjusting *p*-values for multiple-tests with the Benjamini–Hochberg method^63^, the test was significant for all of these three datasets: the TRANSCRIPT ($H=26.5$), PREDICT (*private* version, $H=50.0$), PREDICT (*public*, $H=17.5$) and DNdataset ($H=45.3$) datasets. Eventually, as mentioned in Table 4, we suggest the evaluation of drug repurposing methods on the private version of PREDICT (if the associated generating code can be run) or on the DNdataset which seem to be the most predictive of the drug-disease associations.

#### As a general rule, matrix-oriented methods perform better, probably due to an evaluation bias  

We now focus on developing future collaborative filtering approaches for drug repurposing. Across the top algorithms for average testing (global) AUC and NS-AUC values in Fig. 3, the frequency of a pair-oriented algorithm being in the Top-3 is only $27/(4 \times 8 \times 3) \approx 28\%$, where the HAN algorithm^55^ is the most frequent top pair-oriented method. This frequency decreases to $25\%$ when considering only the top contender, whereas 36% of the algorithms in Table 2 are pair-oriented. Alas, the reason behind this is probably a certain amount of data leakage happening due to the structure of matrix-oriented methods, as described in the problem statement. As such, even though this group of algorithms has good performances, we advise focusing on pair-oriented algorithms for Question 3 in Table 4. One solution to overcome this bias when evaluating a matrix-oriented algorithm might be to ensure only known associations are present in the testing set and then to run evaluation metrics only on these known associations. However, it might still be an unsatisfying solution, as the number of known associations in drug repurposing is extremely small, as illustrated by Table 1.

#### General-purpose collaborative filtering algorithms remain competitive  

Some of the algorithms present in Table 2 were not explicitly developed for drug repurposing but aimed to provide a generic recommender system for various goals, for instance, movie recommendation. As those algorithms are often ignored in drug repurposing-focused publications, we selected some general-purpose algorithms for the benchmark: based on matrix factorization approaches (ALS-WR, LibMF, LogisticMF, PMF) or embedding learning with neural networks (Fast.ai implementation of a collaborative learner). Our benchmark shows that those methods remain competitive for the drug repurposing problem, particularly LogisticMF, even if they are often not the top contender. As such, we advocate for including a comparable general-purpose recommender system when evaluating the performance of a drug repurposing algorithm. Moreover, somehow counterintuitively, the hybrid collaborative filtering algorithms, that is, those that leverage drug and disease features, are not necessarily better than the pure collaborative filtering ones. For instance, LogisticMF, and sometimes the collaborative filtering algorithm from Fast.ai, are among the top contenders in AUC and NS-AUC on all datasets for random or weakly correlated splits in Fig. 3. More often than not, it turns out that being “matrix-oriented” (and the corresponding bias that we discuss above) is more critical for performance than leveraging the features.

#### Neural networks are noticeably better at generalizing  

We observed the influence of weakly correlated training and testing subsets on the performance of models. From Fig. 3, we expect that the difference in performance is vast between random and weakly correlated training and testing sets, independently from the validation metric and the algorithm. To confirm or infirm this assumption, we tested with a Kruskal-Wallis H test whether the median testing NS-AUC value across all datasets is significantly different for a specific type of algorithm (matrix factorization, neural networks, graph-based) on random splits compared to weakly correlated splits. It turns out that the difference in median values is significative at level $\alpha =1\%$ (with *p*-values adjusted for multiple tests) for all types of algorithms and yields respective H-values 21.4, 308.5 and 1, 100.2 for neural networks, graph-based approaches, and matrix factorization methods. The lower the H-value is, the lesser the difference in performance when facing a testing subset weakly correlated to the training data. Unsurprisingly, neural networks are shown to have the most significant ability to generalize and be robust under data distribution shifts, which seems on par with observations from other research fields^64^. However, graph-based approaches come second.

**Table 6:** Results of Kruskal–Wallis H-tests for each dataset. For a fixed dataset *d*, the null hypothesis is “the median NS-AUC value $\mu _\text {NN}(d)$ obtained on dataset *d* by pair-oriented neural networks is equal to the median NS-AUC value $\mu _\text {GB}(d)$ on the same dataset by pair-oriented graph-based approaches”. In each test, the number of elements in each group is $N=200$. The values are rounded up to the closest first or second decimal places. All tests on adjusted *p*-values are significant at level $\alpha =1\%.$.

Dataset	Cdataset	LRSSL	PREDICT	DNdataset	TRANSCRIPT	Fdataset	PREDICT (*public*)	Gottlieb
H	26.4	43.0	70.8	84.1	97.3	128.5	143.2	144.1
$\mu _{NN}-\mu _{GB}$	$-0.07$	$-0.06$	$-0.09$	$-0.21$	$-0.08$	$-0.11$	$-0.11$	$-0.11$

#### Graph-based approaches perform best  

Given our previous remarks, we restrict our comparison of algorithm types to pair-oriented methods. This automatically excludes matrix factorization approaches in our benchmark, according to Table 2. For each dataset, we want to determine whether a specific type of drug repurposing is noticeably better than the other. Similarly to our previous tests, we compare the median validation metric obtained by neural networks and graph-based approaches. The result table is shown in Table 6. Overall, graph-based approaches have a performance significantly superior to neural networks. We suppose that since most of these graph-based approaches aim to reconstruct a graph connecting drugs and diseases (including edges between pairs of drugs or diseases), these methods might be able to uncover some form of reasoning behind a given drug–disease association. Since graph-based methods have some ability to generalize, we recommend developing further the idea of completing drug–disease heterogeneous graphs for drug repurposing.

## Discussion

To better understand the current landscape in collaborative filtering-based drug repurposing, we developed a benchmark of the 11 pure and hybrid collaborative filtering algorithms present in Table 2 on several diverse datasets shown in Table 1. We focused on the validation metrics mentioned in Table 3. This extensive benchmark allowed us to answer important questions about the proper development and evaluation of such models, especially related to their end goal: drug repurposing. Overall, we showed that specific care should be brought to the design and testing of drug repurposing models, as mistakes might lead to biased evaluations. We suggest developing further graph-based methods, which are promising according to our benchmark. Due to the scarcity of the datasets, finer hyperparameter selection across datasets is difficult. However, it would allow us to strengthen our findings in this large-scale benchmark. Moreover, the LRSSL and PREDICT datasets have missing values. In that case, we applied a simple imputation method with the average feature value (described in the methods). Even if this approach is shown to have a good empirical performance on real-life datasets^65,66^, testing other imputation approaches might more significantly validate our findings. Finally, even though there seems to be a correlation between low sparsity number *s* and high classification performance on the dataset, the fact that the least sparse dataset TRANSCRIPT is also the hardest shows that there is more to it. Investigating this lead would constitute an interesting subsequent work. Nonetheless, we hope that those contributions and insights will further improve the development and the real-life application of drug repurposing approaches.

We have identified several future works of interest in this field of research. First, in addition to the prediction of novel drug–disease associations, an application in practice for medical purposes needs the implementation of accountability, meaning that further arguments beyond a simple score should be provided to justify a predicted positive association. The increase in the research related to interpretable or explainable machine learning is a step toward tackling this issue. Moreover, actual prediction scores can rank and prioritize specific drug–disease associations but do not represent a probability or an actual meaningful quantification of the strength of the association. Being able to quantify accurately and control for errors in false positive associations, for instance, is another important venue for research, related to the problem of calibration^67^. Finally, the problem of missing values is pervasive in many research fields, and biology is no exception. Whether imputation methods should be specific to biological data types is an interesting question, especially in the context of preserving interpretability and good calibration.

## Methods

We describe in this section supplementary details about the benchmark and the statistical tests applied in the paper.

**Fig. 4:**
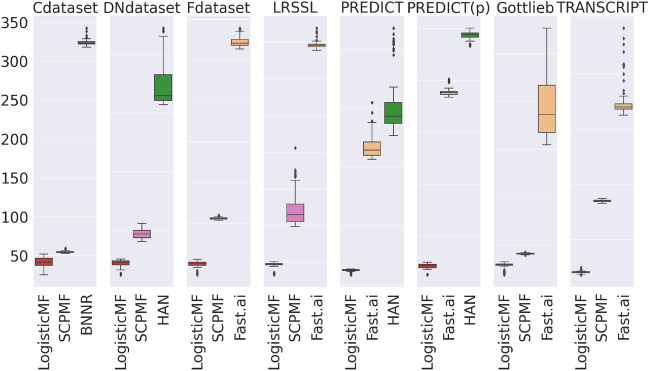
Training times in seconds across $N=100$ iterations for each dataset and the fastest three algorithms among the most frequent Top-3 reported in Figure 3.

### Selection of state-of-the-art algorithms  

We have considered drug repurposing algorithms from the recent literature (less than 8-year-old), which were: 1. based on collaborative filtering, 2. using as input only three matrices, as described in the problem statement, 3. implemented and their code available in open-source or in a readily executable binary file. As such, all algorithms that we considered were run with their original implementation in R, MATLAB/Octave, or Python. In some cases, they encountered errors during their run. Please refer to the benchmark status in Table 9. A reimplementation in pure Python would probably fix these errors. However, this work is out of the scope of our paper. We also report in Fig. 4 for each dataset the boxplots of training times (*i*.*e*., the time to perform a 5-fold cross-validation) for the fastest three algorithms among those reported in at least two Top-3 in Fig. 3.

The prediction times (*i*.*e*., the time to generate scores on the $20\%$ remaining drug–disease associations) are of the order of the second on all datasets and most algorithms. The exceptions are Fast.ai^52^ and NIMCGCN^53^, where the maximum prediction time across iterations and datasets is at most 50 seconds.

### Processing of missing data in the benchmark  

Missing data refers here to unknown values in drug and disease feature matrices *S* and *P*, and occurs in dataset PREDICT (in the private version, 22% of drug feature values are missing in *S*, and around 83% in *P*). To deal with this, for any dataset and any algorithm, each missing feature is imputed by the average value across the corresponding line (that is, other values for the same feature type across the dataset), and then standard-centered with classes SimpleImputer and StandardScaler in scikit-learn^68^ before training a model.

### About the Negative-Sampling AUC (NS-AUC) metric  

**Fig. 5:**
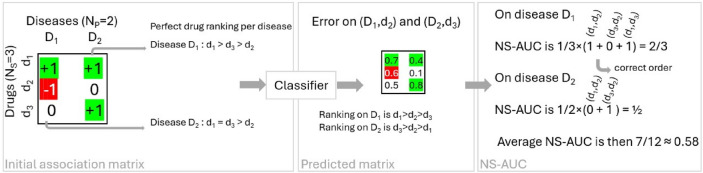
Illustration of the computation of the NS-AUC on an instance with $N_S=3$ drugs and $N_P=2$ diseases.

As described in the original paper^60^, the Negative Sampling-AUC (NS-AUC) is a ranking measure related to the frequency of correctly ranked item (drug) pairs at a fixed user (disease). An example of the computation of the NS-AUC metric is shown in Fig. 5.

### Weakly correlated splits  

We introduced a simple procedure that generalizes the principle of assessing the predictive power of a model on novel drugs, dissimilar to the ones present in the training subset^34^. In prior works, authors chose a simple thresholding criterion, where drugs present in training and testing subsets have a Tanimoto similarity score on chemical structures at most 0.80.

**Fig. 6:**
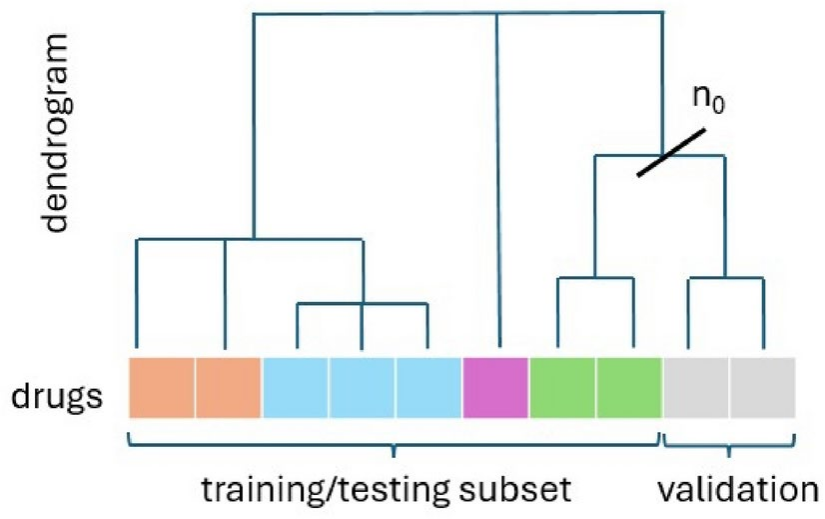
Illustration of the “weakly correlated” splitting approach to obtain training and validation subsets from a dataset.

Given a parameter $s \in (0,1)$ corresponding to the desired percentage of associations in the training set, our procedure automatically splits the dataset of associations into two subsets such that the cosine similarity (by default) in a pair of drugs from different subsets is small. Our algorithm leverages a dendrogram built from a hierarchical clustering (with average linkage) applied to the drug feature vectors. Then, the procedure identifies with binary search the number of clusters $n_0$, $2 \le n_0 \le N_S$, such that there exists a cluster identifier $c_0 \le n_0$

$$\begin{aligned} |\{(d, p) \in A \mid \text {Clust}(d) \le c\}| \approx (1-s) N_S N_F\;, \end{aligned}$$

where $\text {Clust}$ is the function that assigns to a drug its cluster identifier in $\{1,2,\dots ,n_0\}$. In Fig. 6, the corresponding number of clusters for $s=80\%$ is $n_0=5$ and $c_0=4$.

This procedure has a cubic time and memory computational complexity in the number of drugs in the worst case. In practice, for the small drug repurposing datasets in this paper, the computational cost of this procedure is negligible compared to the training phase.

**Table 7:** The average ± standard deviation validation metric on the randomly selected testing subset across $N=100$ iterations for the Top-10 algorithms on the “Synthetic” dataset in Table 1. Average (resp., standard deviation) values are rounded to the closest second (resp., first) decimal place.

Model	NS AUC	AUC
HAN	$1.00 \pm 0.0$	$1.00 \pm 0.0$
BNNR	$1.00 \pm 0.0$	$1.00 \pm 0.0$
LogisticMF	$1.00 \pm 0.0$	$1.00 \pm 0.0$
ALSWR	$1.00 \pm 2.10^{-6}$	$1.00 \pm 1.10^{-3}$
Fast.ai	$1.00 \pm 1.10^{-2}$	$1.00 \pm 1.10^{-3}$
LibMF	–	$0.95 \pm 9.10^{-4}$
PMF	$0.99 \pm 2.10^{-3}$	$0.93 \pm 4.10^{-3}$
SCPMF	$0.88 \pm 2.10^{-1}$	–
NIMCGCN	$0.54 \pm 4.10^{-3}$	$0.94 \pm 5.10^{-4}$

**Table 8:** The average ± standard deviation validation metric on the weakly correlated testing subset across $N=100$ iterations for the Top-10 algorithms on the “Synthetic” dataset in Table 1. Average (resp., standard deviation) values are rounded up to the closest second (resp., first) decimal place.

Model	NS AUC	AUC
HAN	$1.00 \pm 0.0$	$1.00 \pm 0.0$
Fast.ai	$1.00 \pm 0.0$	$1.00 \pm 9.10^{-4}$
LogisticMF	$0.99 \pm 9.10^{-4}$	$0.99 \pm 1.10^{-4}$
BNNR	$0.76 \pm 3.10^{-3}$	$0.98 \pm 2.10^{-4}$
NIMCGCN	$0.54 \pm 3.10^{-4}$	$0.97 \pm 4.10^{-6}$
ALSWR	$0.50 \pm 0.0$	–
LibMF	$0.45 \pm 1.10^{-16}$	$0.98 \pm 3.10^{-16}$
SCPMF	$0.44 \pm 7.10^{-2}$	$0.40 \pm 1.10^{-1}$
LRSSL	–	$0.15 \pm 8.10^{-3}$
PMF	–	$0.08 \pm 7.10^{-3}$

### Synthetic dataset  

The synthetic dataset in Table 1 is the only dataset not directly available from the literature. It allows us to define a task with a controllable level of difficulty. In particular, the synthetic dataset in our benchmark should be an easy task on which all drug repurposing methods should perform excellently and provide a control for some statistical tests.

The generating function takes as input $n_\text {pos}$, the number of positive associations ($+1$’s in matrix *A*), $n_\text {neg}$, the number of negative associations ($-1$’s in matrix *A*), $n_F$, the even number of drug and disease features, and $\mu$, $\sigma$ the parameters from the Gaussian distribution of feature values. In practice, $\mu =0.5$ and $\sigma =1$. Then, we draw each feature value independently and identically (iid) from two Gaussian distributions of mean $\mu$ and $-\mu$ and variance $\sigma ^2$. That is, for any drug or disease $j \le n_\text {pos}, n_\text {neg}$ and feature $i \le n_F$:

$$\begin{aligned} (X_\text {pos})_{i,j} \sim _\text {iid} \mathcal {N}(+\mu , \sigma ) \text { and } (X_\text {neg})_{i,j} \sim _\text {iid} \mathcal {N}(-\mu , \sigma ). \end{aligned}$$

From those matrices, we build the final dataset as follows. *A* is the matrix in $\{-1,0,+1\}^{N_S \times N_P}$ with zeros everywhere except in the square $\{ (i,j) \mid 0 \le i,j \le n_\text {pos}-1\}$ where there is only $+1$, and in the square $\{ (i,j) \mid n_\text {pos} \le i,j \le n_\text {pos}+n_\text {neg}-1 \}$, which only contains $-1$, and where $N_S=N_P=n_\text {pos}+n_\text {neg}$. Then

$$\begin{aligned} S = \begin{bmatrix} (X_\text {pos})_{0\text{ to }N_F-1,\cdot} \\ (X_\text {neg})_{0\text{ to }N_F-1,\cdot} \end{bmatrix} \text { and } P = \begin{bmatrix} (X_\text {pos})_{N_F\text{ to }n_F,\cdot} \\ (X_\text {neg})_{N_F\text{ to }n_F,\cdot} \end{bmatrix} , \end{aligned}$$

where $N_F = n_F/Z \text{ and } M_{k\text{ to }l,\cdot}$ is the matrix where only the rows *k*, $k+1$, $\dots$, $l-1$ to *l* (included) remain. Then, the difficulty of the underlying drug repurposing problem can be tuned by the parameters of the Gaussian distributions $\mu$ and $\sigma$. The larger $\mu > 0$ and the smaller $\sigma$, the easier the problem. See Table 7, resp. Table 8, for the resulting validation matrics on the Top-10 algorithms for random, resp. weakly correlated, training/validation splits.

**Fig. 7:**
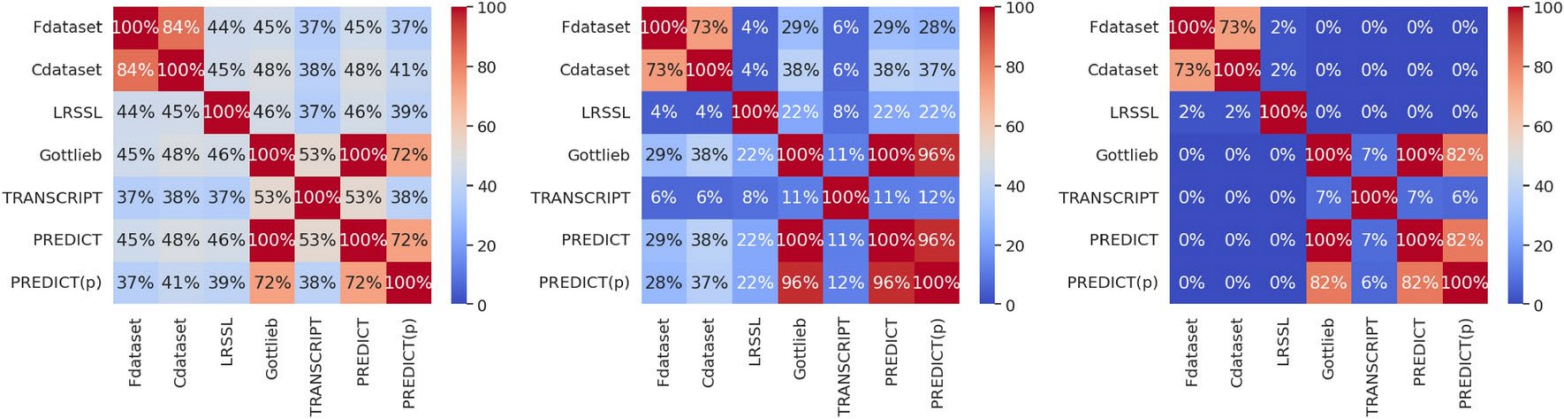
Overlaps, as the size of the intersection over the size of the union multiplied by 100, between drug repurposing datasets listed in Table 1. The left-hand plot is on the list of drugs in a pair of datasets, the center plot represents overlaps for the list of diseases, and the right-hand plot counts the overlaps for the set of positive drug-disease associations.

### Supplementary information about the drug repurposing datasets  

We report overlaps between drug repurposing datasets on Fig. 7. DNdataset does not include drug and disease names, only the contents of the related matrices. Whenever possible, we converted all disease identifiers to MedGen Concept IDs^69^ (if there was no MedGen Concept ID for a disease, we looked for its OMIM identifiers^70^), and all drug identifiers to DrugBank IDs^71^, and, if absent, to PubChem CIDs^72^. Unsurprisingly, there are two rough clusters, one with Fdataset, Cdataset and LRSSL and another with Gottlieb, TRANSCRIPT and PREDICT (private and public versions). As described in the problem statement, Fdataset, Cdataset, and LRSSL use the same drug and disease features.

### Computational resources  

The experiments were run on remote cluster servers of Inria Saclay (processor QEMU Virtual v2.5+, 48 cores @2.20GHz, RAM 500GB) and SBI Rostock (processor Intel Core i7-8750H, 20 cores @2.50GHz, RAM 7.7GB). The clusters of Inria Saclay were favored for pure Python drug repurposing algorithms, whereas the server of SBI Rostock ran the other types of experiments. No GPU was used during the benchmark.

### Benchmark status

Table 9 displays the status of each runs of 100 iterations for each algorithm and dataset in the benchmark.

**Table 9:** Report of the benchmark status across datasets and algorithms. $\checkmark$ means that the 100 iterations were successfully run, whereas $\times$ indicates an error (M: memory, E: runtime error).

Dataset	Split.	ALSWR	LibMF	LogisticMF	PMF	SCPMF	Fast.ai	NIMCGCN	BNNR	DRRS	HAN	LRSSL
Cdataset	Random	$\checkmark$	$\checkmark$	$\checkmark$	$\checkmark$	$\checkmark$	$\checkmark$	$\checkmark$	$\checkmark$	$\checkmark$	$\checkmark$	$\checkmark$
	Weakly c.	$\checkmark$	$\checkmark$	$\checkmark$	$\checkmark$	$\checkmark$	$\checkmark$	$\checkmark$	$\checkmark$	$\checkmark$	$\checkmark$	$\checkmark$
Fdataset	Random	$\checkmark$	$\checkmark$	$\checkmark$	$\checkmark$	$\checkmark$	$\checkmark$	$\checkmark$	$\checkmark$	$\checkmark$	$\checkmark$	$\checkmark$
	Weakly c.	$\checkmark$	$\checkmark$	$\checkmark$	$\checkmark$	$\checkmark$	$\checkmark$	$\checkmark$	$\checkmark$	$\checkmark$	$\checkmark$	$\checkmark$
DNdataset	Random	$\times \text { (M)}$	$\checkmark$	$\checkmark$	$\checkmark$	$\checkmark$	$\checkmark$	$\times \text { (M)}$	$\times \text { (M)}$	$\times \text { (M)}$	$\checkmark$	$\checkmark$
	Weakly c.	$\times \text { (M)}$	$\checkmark$	$\checkmark$	$\checkmark$	$\checkmark$	$\checkmark$	$\checkmark$	$\times \text { (M)}$	$\times \text { (M)}$	$\checkmark$	$\checkmark$
Gottlieb	Random	$\checkmark$	$\checkmark$	$\checkmark$	$\checkmark$	$\checkmark$	$\checkmark$	$\checkmark$	$\checkmark$	$\checkmark$	$\checkmark$	$\checkmark$
	Weakly c.	$\checkmark$	$\checkmark$	$\checkmark$	$\checkmark$	$\checkmark$	$\checkmark$	$\checkmark$	$\checkmark$	$\checkmark$	$\checkmark$	$\checkmark$
LRSSL	Random	$\checkmark$	$\checkmark$	$\checkmark$	$\checkmark$	$\checkmark$	$\checkmark$	$\checkmark$	$\checkmark$	$\checkmark$	$\checkmark$	$\checkmark$
	Weakly c.	$\checkmark$	$\checkmark$	$\checkmark$	$\checkmark$	$\checkmark$	$\checkmark$	$\checkmark$	$\checkmark$	$\checkmark$	$\checkmark$	$\checkmark$
PREDICT (*private*)	Random	$\checkmark$	$\checkmark$	$\checkmark$	$\checkmark$	$\times \text { (E)}$	$\checkmark$	$\checkmark$	$\checkmark$	$\times \text { (E)}$	$\checkmark$	$\checkmark$
	Weakly c.	$\checkmark$	$\checkmark$	$\checkmark$	$\checkmark$	$\times \text { (E)}$	$\checkmark$	$\checkmark$	$\checkmark$	$\times \text { (E)}$	$\checkmark$	$\checkmark$
PREDICT (*public*)	Random	$\checkmark$	$\checkmark$	$\checkmark$	$\checkmark$	$\times \text { (E)}$	$\checkmark$	$\checkmark$	$\checkmark$	$\times \text { (E)}$	$\checkmark$	$\checkmark$
	Weakly c.	$\checkmark$	$\checkmark$	$\checkmark$	$\checkmark$	$\times \text { (E)}$	$\checkmark$	$\checkmark$	$\checkmark$	$\times \text { (E)}$	$\checkmark$	$\checkmark$
TRANSCRIPT	Random	$\checkmark$	$\checkmark$	$\checkmark$	$\checkmark$	$\checkmark$	$\checkmark$	$\checkmark$	$\checkmark$	$\times \text { (E)}$	$\checkmark$	$\times \text { (E)}$
	Weakly c.	$\checkmark$	$\checkmark$	$\checkmark$	$\checkmark$	$\checkmark$	$\checkmark$	$\checkmark$	$\checkmark$	$\times \text { (E)}$	$\checkmark$	$\times \text { (E)}$
Synthetic	Random	$\checkmark$	$\checkmark$	$\checkmark$	$\checkmark$	$\checkmark$	$\checkmark$	$\checkmark$	$\checkmark$	$\times \text { (E)}$	$\checkmark$	$\checkmark$
	Weakly c.	$\checkmark$	$\checkmark$	$\checkmark$	$\checkmark$	$\checkmark$	$\checkmark$	$\checkmark$	$\checkmark$	$\times \text { (E)}$	$\checkmark$	$\checkmark$

### Statistical information  

We report here the missing result tables corresponding to the two-tailed Kruskal–Wallis H-tests run in the experimental study.

#### Predictive power of features in datasets TRANSCRIPT, PREDICT and DNdataset  

Table 10 shows the result table for the corresponding Kruskal–Wallis H-tests. For a fixed dataset *d*, the null hypothesis is “the median NS-AUC value $\mu _\text {wf}(d)$ obtained on dataset *d* by feature-aware methods is equal to the median NS-AUC value $\mu _\text {wof}(d)$ on the same dataset by feature-oblivious approaches. In each test, the number of elements in each group is $N=600$. The values are rounded up to the closest first or second decimal places. The level of significance is $\alpha =1\%$.

**Table 10:** Kruskal–Wallis H-tests on the predictive power of features in datasets A=TRANSCRIPT, PREDICT (B=public and C=private versions) and D=DNdataset. The significance level is set to $1\%$, and *p*-values are adjusted for multiple tests with the Benjamini–Hochberg method^63^. All tests are statistically significant.

Dataset	A	B	C	D
H	26.5	17.5	50.0	45.3
adjusted *p*	0.0	$3.10^{-6}$	0.0	0.0
$\mu _{wf}-\mu _{wof}$	0.07	0.12	0.12	0.14

#### Generalization power of algorithm types  

For a given algorithm type *t*, the null hypothesis is “the median NS-AUC value $\mu _\text {t,Rand}$ obtained by algorithms of type *t* on randomly split training/validation subsets is equal to the median NS-AUC value $\mu _\text {t,WC}$ on weakly correlated subsets. The values are rounded up to the closest first or second decimal places. The level of significance is $\alpha =1\%$. In Table 11, $N_\text {Rand}$, resp. $N_\text {WC}$, is the number of samples in the “random”, resp. “weakly correlated”, group of validation metrics.

**Table 11:** Kruskal–Wallis H-tests on the generalization power of algorithm types “matrix factorization” (MF), “neural networks” (NN) and “graph-based” (GB) across datasets. The significance level is set to $1\%$, and *p*-values are adjusted for multiple tests with the Benjamini-Hochberg method^63^. All tests are statistically significant.

Type	GB	MF	NN
H	308.5	1100.2	21.4
adjusted *p*	0.0	0.0	$4.10^{-6}$
$\mu _{t,Rand}-\mu _\text {t,WC}$	0.10	0.15	0.02
$N_{Rand}$	2500	4600	1700
$N_{WC}$	2500	4600	1800

**Table 12:** Hyperparameters of matrix factorization algorithms.

Model	Hyperparameter	Value
ALSWR	reg	0.01
	alpha	15
	n_iters	15
	n_factors	15
LibMF	fun	0
	k	8
	nr_bins	26
	n_iters	20
	lambda_p1	0.04
	lambda_p2	0.0
	lambda_q1	0.04
	lambda_q2	0.0
	eta	0.1
	do_nmf	False
LogisticMF	num_factors	2
	reg_param	0.6
	gamma	1.0
	iterations	30
PMF	reg	0.01
	learning_rate	0.1
	n_iters	160
	n_factors	15
	batch_size	100
SCPMF	r	15

### Hyperparameter tuning  

We considered for each algorithm the parameters provided in experiments in their current implementation, as, first, most were tested on the text-mining datasets in Table 1 and we aimed to reproduce their results; second, we wanted an evaluation of their performance in “real-life conditions” of drug repurposing, where the hyperparameter tuning is unlikely to be thorough. We were also wary of introducing further data leakage into the benchmark, especially, as the considered drug repurposing datasets are quite small. For general-purpose algorithms, we tune hyperparameters to corresponding values in drug repurposing algorithms, when possible (for instance, the learning rate or the embedding dimension). We report in Tables 12, 13 and 14 below the hyperparameter configurations for each algorithm across all datasets and iterations. We use the same parameter names as in the implementation in the benchscofi package^32^.

**Table 13:** Hyperparameters of neural networks.

Model	Hyperparameter	Value
Fast.ai	n_iterations	5
	n_factors	20
	weight_decay	0.1
	learning_rate	0.005
NIMCGCN	epoch	10
	alpha	10
	fg	256
	fd	256
	k	32
	learning_rate	0.001

**Table 14:** Hyperparameters of graph-based approaches.

Model	Hyperparameter	Value
BNNR	maxiter	300
	alpha	1
	beta	10
	tol1	0.002
	tol2	$1.10^{-5}$
DRRS	–	–
HAN	k	15
	learning_rate	0.001
	epoch	1000
	weight_decay	0.0
LRSSL	k	10
	mu	0.01
	lam	0.01
	gam	2
	tol	0.01
	maxiter	500

## Data availability

### Datasets & algorithms

In addition to repositories mentioned in the publications in which they were introduced, all the datasets mentioned in Table 1 and drug repurposing algorithms in Table 2 are publicly available through the open-source Python packages stanscofi (version 2.0.1) and benchscofi (version 2.0.0)^32^ which can be downloaded from the Python Package Index (PyPI). The only exception is the private version of PREDICT, which cannot be shared freely due to copyright issues with some of the databases on which it was built^14^. Nonetheless, this dataset can be built from scratch from Jupyter notebooks in the following GitHub repository: RECeSS-EU-Project/drug-repurposing-datasets

### Benchmark traces

The results (metrics and runtimes) obtained on each successful iteration of the algorithms and datasets in this benchmark are stored in this GitHub repository: RECeSS-EU-Project/benchmark-results

### Availability of computer code

The implementation of the benchmark pipeline and analysis scripts is publicly available at the following GitHub repository: RECeSS-EU-Project/benchmark-code

After installation and running the benchmark (corresponding instructions are present in the description of the repository), the script generating the figures and the statistical tests in our paper can be run with the following command python3 -m analyses
